# Sedation Is Associated with Higher Polyp and Adenoma Detection Rates during Colonoscopy: A Retrospective Cohort Study

**DOI:** 10.1155/2023/1172478

**Published:** 2023-02-18

**Authors:** Chenghu Xu, Dehua Tang, Ying Xie, Muhan Ni, Min Chen, Yonghua Shen, Xiaotan Dou, Lin Zhou, Guifang Xu, Lei Wang, Ying Lv, Shu Zhang, Xiaoping Zou

**Affiliations:** ^1^Department of Gastroenterology, Nanjing Drum Tower Hospital, The Affiliated Hospital of Nanjing University Medical School, Nanjing, Jiangsu 210008, China; ^2^Jiangsu Medical Quality Control Center (Digestive Diseases), Nanjing, Jiangsu 210008, China; ^3^Department of Gastroenterology, Taikang Xianlin Drum Tower Hospital, The Affiliated Hospital of Nanjing University Medical School, Nanjing, Jiangsu 210046, China

## Abstract

**Background and Aims:**

Currently sedation is a common practice in colonoscopy to reduce pain of patients and improve the operator satisfaction, whereas its impact on examination quality, especially adenoma detection rate (ADR) is still controversial. Thus, we aimed to investigate the association of sedation with ADR.

**Methods:**

Consecutive patients receiving colonoscopy between January 2017 and January 2020 at the Nanjing Drum Tower Hospital, Nanjing, China, were collected. Univariate and multivariate logistic regression models were performed to investigate the association between sedation and ADR. Subgroup analysis and propensity score matching (PSM) analysis, as sensitivity analysis, were performed to validate the independent effect.

**Results:**

The ADR was significantly higher in cases with sedation (ADR: 36.9% vs. 29.1%, odds ratio [OR]: 1.42, 95% confidence interval [CI]: 1.31–1.55, *P* < 0.001). Multivariate analysis showed that the sedation was an independent factor associated with ADR (OR: 1.49, 95% CI: 1.35–1.65, *P* < 0.001). The effect was consistent in subgroup analyses (*P* > 0.05) and PSM analysis (ADR: 37.6% vs. 29.1%, OR: 1.47, 95% CI: 1.33–1.63, *P* < 0.001).

**Conclusion:**

Sedation was associated with a higher polyp and ADR s during colonoscopy, which can promote the quality of colonoscopy.

## 1. Introduction

Colorectal cancer (CRC) is the third most common type of cancer and the second most common cause of cancer-associated mortality over the world [1, 2]. The prognosis of CRC remains poor in advanced-stage cancer, with the overall 5-year survival rate being less than 20%, whereas the early-stage cancer is approximately 90%. As the most effective measures to screening CRC, colonoscopy can detect high-risk lesions of colon cancer and significantly reduce the occurrence of CRC to improving the prognosis of colon cancer, and its inspection quality can be measured by some quality indicators [3]. The adenoma detection rate (ADR) is established as a quality indicator, and an increased ADR has been reported to be related to a reduced risk of interval CRC and mortality [4].

Sedation has become a common practice in endoscopic operation over the past decades [5–7]. Several previous studies have shown that sedation could reduce pain of patients and improve the operator satisfaction [8–11], thus shortened the time of colonoscopy. Controversy surrounding the effect of sedation on quality indicator of colonoscopy, such as ADR had been reported in different country, yet little is known about the impact of sedation when controlling the other factors with a large-scale cohort. Although, some previous studies have reported that sedation has little effect on the ADR of colonoscopy [5, 12–14], some studies recently demonstrating the positive effects of sedation on colonoscopy [6, 7, 15–18]. Therefore, more studies are needed to evaluate whether the sedation is beneficial to ADR.

The aim of this study was to investigate the impact of sedation on polyp and ADR s during colonoscopy. The logistic regression model, subgroup analyses, and propensity score matching (PSM) were used to evaluate and validate the independent effect.

## 2. Methods

### 2.1. Study Design and Patients

This retrospective cohort study collected consecutive patients aged 45–65 years who underwent colonoscopy from January 2017 to January 2020 in Nanjing Drum Tower Hospital (Nanjing, China). The exclusion criteria were as follows: (1) history of colonic resection or CRC; (2) inflammatory bowel diseases and polyposis syndromes; (3) family history of CRC; (4) surveillance; urgent or intent therapeutic colonoscopy; (5) do not reach the cecum or terminal ileum; and (6) invalid withdrawal time and bowel preparation quality. This study has been approved by the Ethics Committee of Nanjing Drum Tower Hospital (DTH-IRB-2021-483-01). As a retrospective study, informed content is not required from participants.

### 2.2. Variables and Measures

The primary outcome was ADR for colonoscopies. ADR was defined as the proportion of colonoscopies in which at least one polyp and adenoma was detected, respectively [14]. Considering the situation that certain polyps need another procedure to remove it, biopsy was unnecessary. Among those colonoscopies without polyps' specimens and marked by endoscopist need to another procedure and the lesions being confirmed as adenoma in 180 days, previous polyp would be treated as an adenoma. All the adenoma tissues were examined histopathologically, reviewed, and confirmed by the pathologists. Advanced adenoma was defined as adenoma of at least 10 mm in size, with more than 25% villous features and/or with high-grade dysplasia [19]. The use of sedation and endoscopic manifestation of polyp were obtained from medical records. The withdrawal time as the time from the cecum identification to the time across the anus [20], was calculated by the program, which can recognize the picture of cecum using the machine learning and the last captured picture and acquire the time from the documents. The Boston Bowel Preparation Scale (BBPS) was used to assess the quality of bowel preparation and quantified by endoscopists during operation [21]. Endoscopist annual volume was determined from the number of colonoscopies performed by endoscopists annually in recent 3 years. Endoscopist experience was defined as years since completing colonoscopy independently. The complaint and diagnosis were collected from medical history. In subgroups and interactions analyses, age categorized as 45–49, 50–59, 60–69, and 70–75 years, annual volume of colonoscopy categorized as <300, 300–700, and >700 per year, and endoscopist experience categorized as <3, 3–7, and >7 years.

### 2.3. Statistical Analysis

Continuous variables with normal distribution were expressed as the mean ± standard deviation (SD). Categorized variables were summarized as counts and proportions. Continuous variables were compared between groups using the Student's *t*-test (normal distribution). Continuous data with non-normal distribution presented as medians and interquartile ranges and compared with. Other categorical variables were compared between groups using the chi-squared test. The univariate logistic regression model was used to investigate the effect size of factors for ADR and the results were presented by odds ratio (OR) and 95% confidence interval (CI). Multivariate logistic regression was further performed to evaluate the association between sedation and ADR in two models adjusting for selected confounding variables. Confounders adjusted in the Model I was selected based on their associations with the outcomes of a change in effect estimate of more than 10%. Model II adjusted all potential confounders. Subgroup and interaction analyses were performed to ensure the stability of the result for sensitivity analysis. PSM analysis was performed as sensitivity analysis in a 1 : 1 ratio to balance the baseline between groups using a greedy nearest neighbor-matching technique. A caliper width of 0.01 of the SD for the logit of the PSM was used for the developed PSM. On matching, six of baseline covariates that could possibly influence the detection rate were used, age, sex, reason for colonoscopy, endoscopist volume, and endoscopist experience. These covariates were acknowledged risk factors of CRCs according to previous studies or influencing factors of quality of colonoscopy. All reported *P-*values were two-tailed, and a CI of 95% was used throughout.

A *P*-value <0.05 was considered statistically significant. All the analyses were performed using the statistical software SPSS version 22.0 (IBM Corporation, Somers, NY, USA).

## 3. Results

### 3.1. Baseline Characteristics

**Figure 1:**
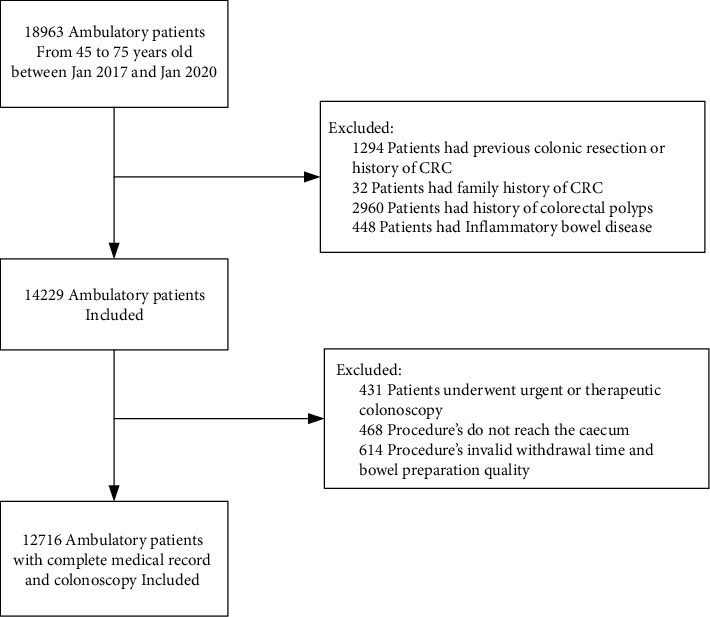
Flow chart for inclusion and exclusion of patients.

Overall, we identified 18,963 ambulatory patients who underwent colonoscopy between January 1, 2017 and January 31, 2020. A total of 6787 cases were excluded according to exclusion criterion. A total of 12,176 patients were finally included for final analysis in our study, of which 9250 (76.0%) patients received sedation (Figure 1).  Table 1 showed the characteristics of the two groups of patients and the comparison results. The mean age (SD) of patients in the no-sedation group was 58.4 (8.1) years, older than the sedation group, which was 57.7 (7.7) years (*P* < 0.001). There was a higher proportion of male in the no-sedation group than sedation group (55.1% vs. 50.1%, *P* < 0.001). 98.8% no-sedation colonoscopies were manipulated in the afternoon. The average withdrawal time was shorter in sedation group (*P* < 0.001). The reasons for colonoscopy, 77.5% were diagnosis, 9.9% was followed by screening in no-sedation group, and compared with sedation group, 67.0% and 19.9%, respectively. The group of sedation colonoscopy has a greater rate of treatment towards polyp.

### 3.2. Outcomes

ADR was higher in group with sedation (ADR: 36.9% vs. 29.1%, *P* < 0.001; Table 2).

### 3.3. The Effect of Sedation and Interaction Effects between Sedation and Other Factors

To explore the effect of the factors on the ADR of the colonoscopy, univariate and multivariate logistic regression analyses were performed. In univariate logistic regression analysis, for patient-level factor, we found that the female patients (OR: 0.53, 95% CI: 0.50–0.57) were negatively associated, whereas age (OR: 1.03, 95% CI: 1.03–1.04), sedation (OR: 1.42, 95% CI: 1.31–1.55), and withdrawal time (OR: 1.14, 95% CI: 1.13–1.15) were positively associated with ADR (Table 3).

Furthermore, for multivariate analyses, we had constructed regression analysis models including crude, Model I included the factors were statistically significant, and Mode II included alls factors we collected. After adjusting for sex, age, time of colonoscopy, withdrawal time, and experience of endoscopist in Model I and all potential confounders in Model II, the association between sedation and ADR was still stable in both models [Model I: ADR (OR: 1.49, 95% CI: [1.35, 1.65], *P* < 0.001); Model II: ADR (OR: 1.48, 95% CI: 1.33–1.63, *P* < 0.001)] (Table 4).

### 3.4. Subgroup and Sensitivity Analysis

**Figure 2:**
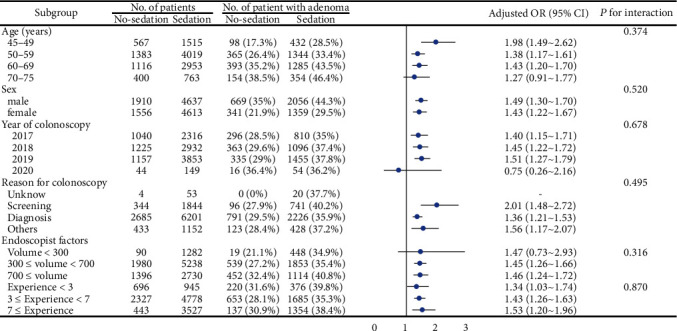
Subgroup analysis of the effect of sedation on ADR. Adjust for: sex; age; year of colonoscopy; time of colonoscopy; reason for colonoscopy; withdrawal time; score of BBPS; volume of endoscopist; and experience of endoscopist.

In the subgroup analysis, there was no apparent interaction between any subgroup (Figure 2). After PS matching, the baseline was well balanced between groups (Table 5). The ADR was higher in the sedation group, whereas withdrawal time was longer than in no-sedation group (ADR: 37.6% vs. 29.1%, OR: 1.47, 95% CI: 1.33–1.63, *P* < 0.001). Comparison between sedation and no-sedation groups after PSM showed that no significant difference was found on detection rate of advanced adenoma (8.2% vs. 7.5%, *P* = 0.280; Table 6).

## 4. Discussion

In this retrospective cohort study, we comprehensively analyzed the impact of sedation on ADR. Our results revealed that after adjusting potential confounding factors, the colonoscopy with sedation was significantly associated with higher ADR. After PSM, the results were still stable, which indicated that sedation was an independent factor associated with a higher ADR [22].

To our knowledge, this is the largest study to date specifically evaluating the effects of sedation in ADR among outpatient colonoscopies. In previous studies, it is reported that sedation can improve patient comfort and satisfaction [12, 16, 23–25]. However, the overall quality indicators, the findings (about impact of sedation) are controversial, both in terms of demonstrating an increase and in terms of demonstrating similarity. Bannert et al. [5] reported that ADR is unaffected by sedation, but without registered level of sedation and type of sedation remain in this study. Nakshabendi et al. [13] recognized that a propofol sedation can lead to detection of more advanced polyps but did not find a difference in ADR in the use of sedation. Krigel et al. [14] found no association between anesthesia assistance and ADR among trainees. Zhao et al. [15] found no help on ADR of sedation, but multivariate analysis for evaluating confounding factors was not performed. Zhang et al. [7] and Huang et al. [17] found that sedation was a favorable factor to improve ADRs, but the history of colorectal disease did not been investigated. Khan et al. [26] also found that sedation as opposed to no sedation was significantly associated with a higher ADR, but only 179 of 24,795 patients underwent unsedated colonoscopies, which was too small to draw robust conclusion. Compared with previous studies, our study had a larger sample size with a screened population, and considerable factors, such as experience of endoscopist, were considered as confounding factors to adjust the effect of sedation on ADR, which makes our outcome more reliable and convinced.

Our hypothesis is that sedated patients have a higher tolerance for endoscopy, and even though the withdrawal itself is painless, the patient's reactions may have an impact on the stability of the endoscope, and sometimes these reactions are unconscious and unrecognized. Adding a paragraph on the effects of propofol and fentanyl on bowel motility, the withdrawal the retraction process is the concentrated search for polyps. A steady and clear picture with continuous focus is helpful for the colonoscopists' ability to find polyps. Even though the retraction process is a relatively painless one, sometimes the endoscope slips, which requires reentry, and this process would be painful in a non-sedated procedure.

However, the relationship between sedation and withdrawal time has rarely been studied. Previous studies have suggested that withdrawal time increases the detection of colonic lesions and thus improves ADR [4, 27–30]. Although with advances in computer and information technology and we can get the cecal identification from machine learning, the time of biopsies, and other operations are still not counted [31]. This makes it difficult to explore the relationship between withdrawal time and sedation, but for specific colonoscopies, such as screening-only colonoscopies, it is comparable. We need to explore fully to validate the relationship between withdrawal time and sedation in the future.

### 4.1. Limitations

First, this study has inherent limitations associated with retrospective data collection, and some potential confounders, such as body mass index, smoking status, alcohol intake, and medication use, may be neglected in the analysis. Thus, further prospective study was needed for qualified data collection and management. Second, biopsy time and polyp removal time were not precisely calculated and subtracted, which leading a redundant withdrawal time. Although we performed subgroup analysis according to the operation in the examination, analysis of withdrawal time for ADR with subgroups of sedation remains to be confirmed by larger random controlled trials or more refined retrospective studies. Third, limitation of our study is that outbalance the discrepancy between sedated and no sedated patients. Patients are free to choose to undergo sedation examinations in our center. Many modalities influence whether a patient chooses sedation, not the least of which is the patient's pursuit of comfort, fear of possible pain. Most of patients in our study were able to tolerate non-sedated colonoscopy, which was safe enough too. In fact, we took into inherent differences account, such as those caused by patients' propensity to choose in the study design, and enrolled ambulatory patients who had their first colonoscopy when sedation be clearly associated with improved patient satisfaction. Of course, a prospective multi-center study with balanced groups is essential to further validate our results.

No single method can be a silver bullet to improve the quality of colonoscopy, and each method of improving endoscopic detection rates is subject to marginal effects. The purpose of our study was to provide a real-world perspective on colonoscopic sedation. The use of sedation can both improve patients' satisfaction and endoscopist performance.

## 5. Conclusion

In conclusion, compared with colonoscopy without sedation, colonoscopy with sedation has a positive effect on ADR. When controlling for other confounding factors, sedation was an independent predictor of higher ADR. From a quality improvement perspective, choosing the sedation procedures in colonoscopies is favorable.

## Figures and Tables

**Table 1:** Characteristics of the patients.

Variable	All patients (*N* = 12,176)	No-sedation (*N* = 3466)	Sedation (*N* = 9250)	*P*-value
Age (years)	57.9 ± 7.8	58.4 ± 8.1	57.7 ± 7.7	<0.001
Sex				
Male	6547 (51.5%)	1910 (55.1%)	4637 (50.1%)	<0.001
Female	6169 (48.5%)	1556 (44.9%)	4613 (49.9%)	
Year of colonoscopy				
2017	3356 (26.4%)	1040 (30%)	2316 (25%)	<0.001
2018	4157 (32.7%)	1225 (35.3%)	2932 (31.7%)	
2019	5010 (39.4%)	1157 (33.4%)	3853 (41.7%)	
2020	193 (1.5%)	44 (1.3%)	149 (1.6%)	
Time of colonoscopy				
AM (8–12)	4282 (33.7%)	42 (1.2%)	4240 (45.8%)	<0.001
PM (12–17)	8434 (66.3%)	3424 (98.8%)	5010 (54.2%)	
Reason for colonoscopy				
Unknown	57 (0.4%)	4 (0.1%)	53 (0.6%)	<0.001
Screening	2188 (17.2%)	344 (9.9%)	1844 (19.9%)	
Diagnosis	8886 (69.9%)	2685 (77.5%)	6201 (67.0%)	
Others	1585 (12.5%)	433 (12.5%)	1152 (12.5%)	
Endoscopist factors				
Volume^a^ < 300	1372 (10.8%)	90 (2.6%)	1282 (13.9%)	<0.001
300 ≤ volume < 700	7218 (56.8%)	1980 (57.1%)	5238 (56.6%)	
700 ≤ volume	4126 (32.4%)	1396 (40.3%)	2730 (29.5%)	
Experience^b^ < 3	1641 (12.9%)	696 (20.1%)	945 (10.2%)	
3 ≤ experience < 7	7105 (55.9%)	2327 (67.1%)	4778 (51.7%)	
7 ≤ experience	3970 (31.2%)	443 (12.8%)	3527 (38.1%)	
Score of BBPS	6.1 ± 1.3	6.0 ± 1.3	6.1 ± 1.3	<0.001
Total number of biopsies	1.2 ± 1.6	1.1 ± 1.6	1.2 ± 1.6	<0.001
Number of polyps per colonoscopy	0.7 ± 0.8	0.6 ± 0.8	0.7 ± 0.8	<0.001
Treatment				
No treatment	5659 (44.5%)	1767 (51%)	3892 (42.1%)	<0.001
Deal with biopsy forceps	5170 (40.7%)	1385 (40%)	3785 (40.9%)	
Polypectomy with other method^c^	1887 (14.8%)	314 (9.1%)	1573 (17%)	
Withdrawal time (minutes)	8.7 ± 4.9	8.8 ± 4.9	8.6 ± 4.9	<0.058
No treatment	7.1 ± 3.6	7.3 ± 3.4	7.0 ± 3.6	
Deal with biopsy forceps	8.3 ± 3.9	8.9 ± 4.0	8.1 ± 3.9	
Polypectomy with other method	14.4 ± 6.5	17.0 ± 7.0	13.9 ± 6.3	
Group of withdrawal time (minutes)				
<6	3250 (25.6%)	786 (22.7%)	2464 (26.6%)	<0.001
6–12n	7202 (56.6%)	2060 (59.4%)	5142 (55.6%)	
>12	2264 (17.8%)	620 (17.9%)	1644 (17.8%)	

BBPS: the Boston Bowel Preparation Scale score.

^a^Volume: the number of colonoscopies performed by endoscopists annually.

^b^Experience: years since completing colonoscopy independently.

^c^Including polypectomy with snare wire, argon plasma coagulation, or electrocoagulation.

**Table 2:** Outcome comparison between sedation and no-sedation groups.

Variable	All patients (*N* = 12,716)	No-sedation (*N* = 3466)	Sedation (*N* = 9250)	*P*-value
ADR, *n* (%)	34.8% (4425)	29.1% (1010)	36.9% (3415)	<0.001

ADR: adenoma detection rate.

**Table 3:** Univariate logistic regression analysis of risk factors for adenoma detection rate.

Variables	OR (95% CI)	*P*-value
Age (years)	1.04 [1.03, 1.04]	<0.001
Sedation		
No	Reference	
Yes	1.42 [1.31, 1.55]	<0.001
Sex		
Male	Reference	
Female	0.53 [0.50, 0.57]	<0.001
Year of colonoscopy		
2017	Reference	
2018	1.10 [1.00, 1.21]	0.052
2019	1.13 [1.03, 1.24]	0.009
2020	1.16 [0.86, 1.57]	0.342
Time of colonoscopy		
Morning (8–12)	Reference	
Afternoon (12–17)	0.82 [0.75, 0.91]	<0.001
Reason for colonoscopy		
Unknown	Reference	
Screening	1.15 [0.66, 1.99]	0.627
Diagnosis	0.95 [0.55, 1.64]	0.857
Others	0.99 [0.57, 1.72]	0.960
Withdrawal time	1.14 [1.13, 1.15]	<0.001
Endoscopist factors		
Volume < 300	Reference	
300 ≤ volume < 700	0.96 [0.85, 1.09]	0.517
700 ≤ volume	1.19 [0.95, 1.21]	0.009
Experience < 3	Reference	
3 ≤ experience < 7	0.86 [0.77, 0.96]	0.008
7 ≤ experience	1.05 [0.85, 0.99]	0.383

**Table 4:** Multivariate logistic regression analysis models evaluating the association between sedation and adenoma detection rate.

Sedation	Crude		Model I		Model II	
	OR [95% CI]	*P*-value	OR [95% CI]	*P*-value	OR [95% CI]	*P*-value
No	Reference		Reference		Reference	
Yes	1.42 [1.31, 1.55]	<0.001	1.49 [1.35, 1.65]	<0.001	1.48 [1.33, 1.63]	<0.001

Model I adjusted for: sex; age; time of colonoscopy; withdrawal time; volume of endoscopist; and experience of endoscopist. Model II adjusted for: sex; age; year of colonoscopy; time of colonoscopy; reason for colonoscopy; withdrawal time; score of BBPS; volume of endoscopist; and experience of endoscopist.

**Table 5:** Characteristics in the full cohort and propensity score-based matched cohort.

	Full cohort		Standardized difference	Matched cohort		Standardized difference	*P*-value
	No-sedation	Sedation		No-sedation	Sedation		
Number of patients	3466	9250		3407	3407		
Age (years)			0.119			0.015	0.941
45–49	567 (16.4)	1515 (16.4)		567 (16.6)	576 (16.9)		
50–59	1383 (39.9)	4019 (43.4)		1369 (40.2)	1344 (39.4)		
60–69	1116 (32.2)	2953 (31.9)		1111 (32.6)	1121 (32.9)		
70–75	400 (11.5)	763 (8.2)		360 (10.6)	366 (10.7)		
Sex, female	1556 (44.9)	4613 (49.9)	0.100	1553 (45.6)	1558 (45.7)	0.003	0.903
Reason for colonoscopy			0.300			0.011	0.976^a^
Unknown	4 (0.1)	53 (0.6)		4 (0.1)	5 (0.1)		
Screening	344 (9.9)	1844 (19.9)		343 (10.1)	335 (9.8)		
Diagnosis	2685 (77.5)	6201 (67.0)		2629 (77.2)	2637 (77.4)		
Others	433 (12.5)	1152 (12.5)		431 (12.7)	430 (12.6)		
Endoscopist factors			0.443			0.042	0.222
Volume^a^ < 300	90 (2.6)	1282 (13.9)		79 (2.3)	59 (1.7)		
300 ≤ volume < 700	1980 (57.1)	5238 (56.6)		1962 (57.6)	1965 (57.7)		
700 ≤ volume	1396 (40.3)	2730 (29.5)		1366 (40.1)	1383 (40.6)		
Experience^b^ < 3	696 (20.1)	945 (10.2)	0.626	665 (19.5)	660 (19.4)	0.023	0.630
3 ≤ experience < 7	2327 (67.1)	4778 (51.7)		2299 (67.5)	2277 (66.8)		
7 ≤ experience	443 (12.8)	3527 (38.1)		443 (13.0)	470 (13.8)		

^a^Volume: the number of colonoscopies performed by endoscopists annually.

^b^Experience: years since completing colonoscopy independently.

**Table 6:** Outcome comparison between sedation and no-sedation groups after PSM.

	No-sedation	Sedation	*P*-value
	(*n* = 3407)	(*n* = 3407)	
ADR, *n* (%)	990 (29.1)	1280 (37.6)	<0.001
AADR, *n* (%)	280 (8.2)	256 (7.5)	0.280
Withdrawal time, minutes (mean ± SD)	8.76 ± 4.83	8.34 ± 4.63	<0.001
Treatment			<0.001
No treatment, *n* (%)	1743 (51.2)	1453 (42.6)	
Deal with biopsy forceps, *n* (%)	1358 (39.9)	1388 (40.7)	
Polypectomy with other method, *n* (%)	306 (9.0)	566 (16.6)	

ADR: adenoma detection rate; AADR: advanced adenoma detection rate.

## Data Availability

Data supporting this research article are available from the corresponding author or first author on reasonable request.
